# Skating Carnival

**Published:** 1868-01

**Authors:** 


### Skating Carnival.

Fifth Avenue, corner of 46th Street, has the best appointments for skating in New-York; this pond is under the personal control of Mr. Alexander McMillan, himself an accomplished skater, and who has at his establishment, 575 Broadway, a complete assortment of New-York Club skates for all sexes and ages; and with the assistance of Mr. J. W. Post of Michigan, who has himself a large experience in the management of skating ponds, McMillan's Fifth Avenue Pond promises to be the place of fashionable resort for the present winter; especially as it has the following very important advantages:

1st It is immediately on the Avenue.

2d The 5th Avenue stages land the skaters on the spot.

3d It is accessible within one block by the 6th Avenue cars, and almost as convenient to the 2d and 3d Avenue lines.

4th The water is on a level clay bottom, at no place deeper than sixteen inches, being regulated by the Croton pipes.

5th The accommodation rooms are at the edge of the ice, and but a foot above it, making them immediately accessible for purposes of adjusting skates, warming, taking refreshments, rest, &c.

6th This building is over one hundred feet long, is abundantly heated and lighted, with separate apartments for Ladies and Gentlemen, besides a large hall in common.

7th The retiring closets will be kept comfortably warmed.

8th Messrs. McMillan and Post will be always on hand to secure a prompt and courteous attendance to all the wants of the visitors.

9th There will be a luncheon room for ladies, with neatly arranged tables for refreshments, of hot coffee, tea, oysters, confections, &c.

There will be approrpriate music from an experienced band every afternoon and evening, with brilliant lights in every direction.

The season opened December 10th, 1867. Season tickets are five dollars each; four dollars for those under 12. Single tickets fifty cents; half price for those under 12.

